# Relationship between posttraumatic growth and help-seeking behavior in use of psychosocial support services among patients with cancer

**DOI:** 10.1007/s11764-023-01418-w

**Published:** 2023-07-19

**Authors:** Tomoko Matsui, Kanako Taku

**Affiliations:** 1 Graduate School of Human Sciences, Osaka University, 1-2 Yamadaoka, Suita City, Osaka Prefecture, Japan; 2 Department of Psychology, Oakland University, Rochester, MI USA

**Keywords:** Patients with cancer, Help-seeking behavior, Posttraumatic growth, Psychosocial support service

## Abstract

**Purpose:**

Cancer survivors who used psychosocial support services often report posttraumatic growth (PTG). This refers to positive psychological changes that may occur as the five domains as a result of the challenges they face. Opposing relationship also might exist. This study aimed to examine the relationship between PTG and help-seeking behavior (HSB).

**Methods:**

In total, 710 participants completed an online survey at Time1. Of those, 395 who reported not using any psychosocial support services at Time1 were asked to participate in the Time2 survey and completed a questionnaire. The participants provided demographic information, the experiences of using psychosocial support services, and the overall and five domains of PTG.

**Results:**

Those who experienced HSB at Time1 reported a higher PTG, and two of the PTG domains, Appreciation of Life and New Possibilities, than those who did not used services. Mixed ANOVAs showed the main effects of the HSB on the overall PTG, Appreciation of Life, and New Possibilities. Hierarchical logistic regression analyses showed that Appreciation of Life at Time1 was significantly related to the engaging in HSB at Time2.

**Conclusion:**

Those who received psychosocial support services reported a higher PTG. Participants may have also engaged in HSB because they had experienced PTG. People who are likely to seek help and experience PTG may share common characteristics.

**Implications for Cancer Survivors:**

Support for those who do not fit the existing PTG and the use of psychosocial support services should also be considered.

## Introduction

Cancer survivors often experience psychological and interpersonal difficulties after diagnosis, in addition to physical problems [1]. Some patients are diagnosed with depression [2] and experience PTSD [3]. Simultaneously, some also experience positive personal changes [4, 5]. It is called posttraumatic growth (PTG) that is defined as positive changes that may occur as a result of a psychological struggle with a highly stressful life event [6]. Generally, PTG consists of five areas: New Possibilities (experiencing as developing a new path or opportunities), Appreciation of Life (experiencing greater appreciation for each day and for the value of life), Relating to Others (positive changes in interpersonal relationships), Existential and Spiritual Change (better understanding of spirituality or religious faith), and Personal Strength (increased self-reliance or greater sense of personal strength) [6–8]. These areas have been quantitatively and qualitatively identified [9, 10]. Related studies on cancer patients have been growing [5, 11], showing that hope, optimism, spirituality, and meaning are factors that contribute to the promotion of PTG [5]. In recent years, PTG trajectories have been reported in cancer survivors [12–14]. Some studies have reported patterns of high or low levels that remain unchanged, whereas others have reported increased or decreased the levels.

The palliation of psychosocial distress in patients with cancer has been emphasized, and a variety of psychosocial support services have become available (e.g., psycho-oncology departments and group therapy). However, few cancer patients use such services, even when they feel severe distress [15, 16]. Help-seeking behavior (HSB) is defined as problem-focused, planned behavior, involving interpersonal interactions with a selected healthcare professional [17]. The number of studies on HSB has also been gradually increasing, and factors contributing to the use of psychosocial support services among patients with cancer include the perceived need for psychosocial services and support, environmental conditions, physical problems, and psychological distress [11, 16, 18–20].

### Complementary relationships between PTG and HSB

The commonality and complementary relationships between PTG and HSB have been highlighted theoretically in terms of culture, such as the positive psychology movement, subjective norms, and personalities at the individual level [11]. Both concepts originated and were refined primarily in the Western cultures. Therefore, people who would seem to fit Western cultural values may be more likely to report PTG and engage in HSB more easily. Meanwhile, we assume that it is difficult for people associated with a culture that is very different from Western values, such as the traditional Japanese culture, to experience both PTG and HSB. For example, previous studies have shown that PTG levels are lower in Japanese samples than in their American counterparts [7, 21]. Other studies have shown that the structure of factors of the Posttraumatic Growth Inventory (PTGI) for Japanese samples was different from the original [22] and that the expansion of the PTGI, the PTGI-X [23], was developed to specifically expand the Spiritual Change of the existing PTGI. As for HSB, its use is approximately 10–15% in Japan [15, 24, 25], compared to approximately 30% in patients with cancer in Western countries [26, 27]. Hence, we hypothesized that people who are more likely to experience PTG are also more likely to demonstrate HSB and that the opposite is also considered to be valid.

### Experiencing HSB may lead to PTG

We hypothesized that experiencing HSB, that is, using psychosocial support services, would result in a higher level of PTG. Some aspects of psychotherapy techniques may promote PTG. Providers of psychosocial support services, psychiatrists, clinical psychologists, and social workers may play the role of expert companions who are well-trained professionals with humble attitudes and can help facilitate PTG [28]. When using psychosocial support services, individuals are required to disclose themselves to others. Increased social support was positively associated with PTG, supporting the notion that social support promotes PTG by enabling the disclosure of a highly stressful event [29]. One article mentioned the elements of psychotherapeutic or psychoeducational interventions for PTG, such as cognitive-behavioral, interpersonal, existential, narrative, and emotion regulation [30]. Additionally, studies examining whether psychosocial interventions, not limited to patients with cancer, can increase PTG levels, have received much attention. One meta-analytical study suggested that current interventions including written/spoken, cognitive-behavioral therapy modestly increase PTG, although the results need to be replicated because of the small number of eligible studies and various types of interventions [31]. Studies have also examined the effects of psychosocial interventions on PTG among patients with cancer. Some studies have shown that those who participated in a group intervention, one was a specific to promoting PTG, overall reported a higher level of PTG [32, 33]. Similarly, a recent meta-analysis focusing on patients with cancer showed that psychosocial interventions including supportive group psychotherapy and multiple health behavior change interventions increased PTG [34].

### Experiencing PTG may lead to HSB

We also hypothesized that PTG experience would lead to HSB, although there is little evidence thus far as most studies have assumed PTG as an outcome. However, people who have experienced trauma and subsequent emotional struggles may have a sense of growth in their relationships, which in turn should help them be more willing to count on others in times of trouble, since it is a domain of PTG—Relating to Others [6, 22]. Talking to others and telling others about oneself are essential for HSB to psychosocial support services. Therefore, it can be assumed that those who have experienced PTG are less likely reserved to seeking help afterwards. One cancer patient who participated in psychosocial support services reported that one of the reasons for participating was because she could not do anything about her anxiety by herself and became to think that it might be fine to talk to someone to get some relief [35]. Additionally, according to one study [8], there may be a common between the three constituent themes (New Possibilities, Appreciation of Life, and Personal Strength), that is, having survived the traumatic event, people felt better at coping with future challenging situations. For example, as these components increase, they may engage in HSB to psychosocial support services as a coping such challenging situations.

We hypothesized that HSB and PTG would be positively associated with each other, as they may have a complementary relationship. This study aimed to examine this relationship using a longitudinal research design among patients with cancer.

## Methodology

### *Procedure and participants (*Fig. 1*)*

**Fig. 1:**
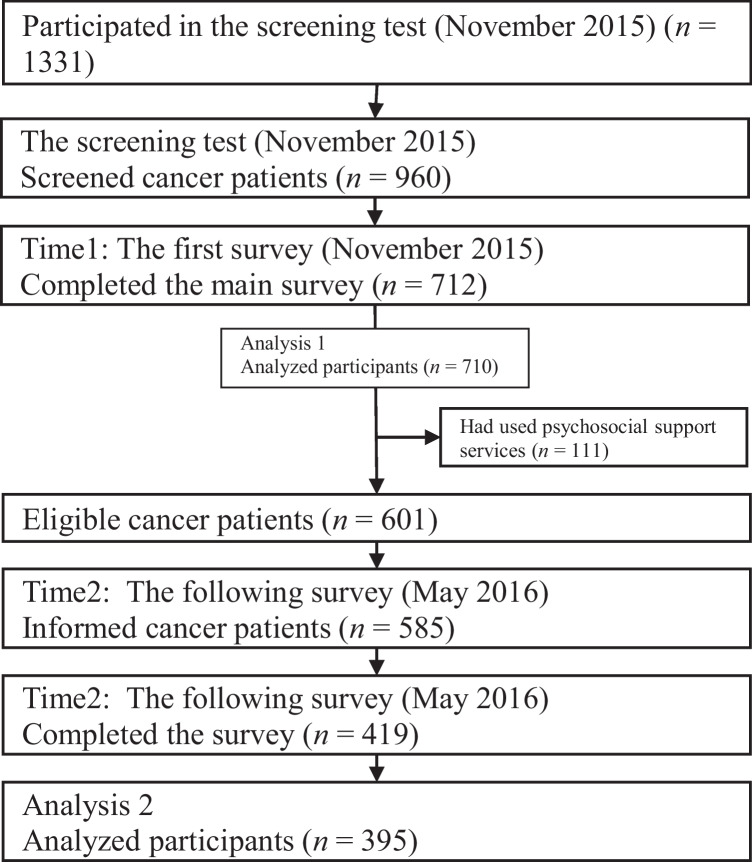
Study flow

We conducted web surveys using a panel of patients who visited a doctor for cancer between July 2014 and July 2015. The data were managed by INTAGE HOLDINGS, Inc., which conducted a voluntarily planned investigation in July 2015 using a questionnaire survey. The company asked respondents about their “illnesses in the past year for which they were prescribed medication at outpatient visits” and “of the illnesses mentioned, those for which they were seeking ongoing consultations.” We used a panel of respondents who mentioned “cancer” as their illness.

Before conducting the Time1 questionnaire survey in November 2015, a screening test was implemented to recruit those who were not hospitalized for more than 5 days at the time of the survey and those who were willing to cooperate for the Time2 survey. This process resulted in 960 eligible participants out of 1331 who took the screening test. Of the 960 participants, 712 completed the Time1 survey, and 710 were analyzed. Of 712,601 people (84.4%) who had never used psychosocial support services before the Time1 survey were eligible to participate in the Time2 survey. In total, 585 participants were asked to participate in the Time2 survey (May 2016), of which 419 responded.

### Measurements

#### Time1 surveys

##### Demographic information, treatment status

Participants’ demographic information including age, sex (male = 1, female = 2), and residential status, was collected.

##### Information on physical status, cancer, and treatment

Details of the participants’ cancer type (multiple answers), treatment status, and date of diagnosis were collected. Additionally, participants were asked to respond to the Karnofsky Performance Scale (KPS), an assessment tool used to measure physical function of cancer patients [36]. They selected one out of six options (see Table 1) in terms of which option best describes their physical condition at the time of the survey (1 = *normal, no complaints* to 6 = *requires considerable assistance*).

**Table 1:** Demographic information

	Analysis 1 (*n* = 710)			Analysis 2 (*n* = 395)	
	*Mean/n*	*SD/%*		*Mean/n*	*SD/%*
*Age*	58.1	11.9		58.8	11.6
*Sex*					
Male	350	49.3		204	51.6
Female	360	50.7		191	48.4
Living alone	95	13.4		51	12.9
Duration since diagnosis (months)	56.7	55.4		59.5	57.4
*Cancer types*					
Breast	201	28.3		104	26.3
Prostate	103	14.5		66	16.7
Colon	90	12.7		50	12.7
Stomach	60	8.5		25	6.3
Lung	34	4.8		24	6.1
Cervical	32	4.5		16	4.1
Blood	24	3.4		16	4.1
Uterine body	24	3.4		9	2.3
Bladder	21	3.0		16	4.1
Liver	18	2.5		11	2.8
Kidney	18	2.5		8	2.0
Ovarian	16	2.3		10	2.5
Brain	6	0.8		5	1.3
Others	128	18.0		76	19.2
*Karnofsky Performance Status*					
Normal, no complaints	407	57.3		242	61.3
Able to carry on normal activities. Minor signs or symptoms of disease	195	27.5		104	26.3
Normal activity with effort	81	11.4		33	8.4
Care for self. Unable to carry on normal activity or to do active work	17	2.4		8	2.0
Requires occasional assistance, but able to care for most of his needs	9	1.3		7	1.8
Requires considerable assistance	1	0.1		1	0.3

The information was obtained Time1

##### The Japanese version of the Hospital Anxiety and Depression Scale [37, 38]

This scale consists of 14 items assessing anxiety and depression scored from 0 to 3 (e.g., “I feel tense or ‘wound up.’” “I still enjoy the things I used to enjoy.”). The total score is recommended for assessing psychological distress, and the recommended cutoff score is 10/11 for adjustment disorder and major depression. Cronbach’s alpha coefficient was 0.91.

##### The Japanese version of the Posttraumatic Growth Inventory (PTGI-J) [22]

This scale consists of 21 items scored from 0 to 5. Participants responded to this inventory by indicating how much they felt they had changed as a result of their cancer diagnosis (e.g., “I can better appreciate each day.” “I changed my priorities about what is important in life.”). Cronbach’s alpha coefficients were as follows: New Possibilities *α* = 0.88, Appreciation of Life *α* = 0.72, Relating to Others = 0.90, Existential and Spiritual Change *α* = 0.53, and Personal Strength *α* = 0.82; and total score of PTGI *α* = 0.95.

##### Information on their use of psychosocial support services

We asked the participants whether they used psychosocial support services to resolve or cope with problems after being diagnosed with cancer. Those who reported having received psychosocial support services were asked to select the services they had used from the list in Table 2.

**Table 2:** Contents of psychosocial support services

	Time1 (*n* = 111)		Time2 (*n* = 21)	
	*n*	*%*	*n*	*%*
Psychosomatic medicine	31	27.9	4	19.0
Cancer consulting and support center	27	24.3	7	33.3
Cancer salon	18	16.2	5	23.8
Medical care counseling	16	14.4	4	19.0
Counseling by therapist	12	10.8	3	14.3
Support group	12	10.8	2	9.5
Psychiatry	11	9.9	1	4.8
Regional medical cooperation office	7	6.3	2	9.5
Psycho-oncology	2	1.8	0	0
Group therapy	1	0.9	0	0
Others	12	10.8	0	0

#### Time2 Survey

##### The PTGI-J [22]

This scale consists of 21 items scored from 0 to 5. Participants responded to this inventory by indicating how much they felt they had changed as a result of their cancer diagnosis. Cronbach’s alpha coefficients were as follows: New Possibilities *α* = 0.89, Appreciation of Life *α* = 0.75, Relating to Others *α* = 0.91, Existential and Spiritual Change *α* = 0.61, and Personal Strength *α* = 0.81; and total score of PTGI *α* = 0.95.

##### Information about the use of psychosocial support services since Time1

We asked participants whether they used psychosocial support services to resolve or cope with problems after being diagnosed with cancer between Time1 and Time2 surveys. Those who had received such services were asked to report the chosen types of services that are listed in Table 2.

### Analysis

The PTGI score was divided by the number of items, and the mean was used as the total score and for each of the five domains. Descriptive statistics were calculated and a correlation analysis was conducted using the total score, and each domain of the PTGI, and the demographic items. *T*-tests were conducted to compare Support Receivers and Support Non-Receivers. After conducting the correlation analysis, mixed ANOVAs were conducted to examine the relationship between PTG and HSB (within-subjects factor: Time1 and Time2 PTG; between-subjects factor: whether participants used psychological support services). Additionally, hierarchical logistic regression analysis was performed with the use of psychosocial support services at Time2 (non-use = 0, use = 1) as a dependent variable, using age and sex as predictors in Step 1 (forced input) and the Time1 five PTG domain scores in Step 2 (variable reduction method) to examine whether PTG at Time1 predicted the use of psychosocial support services. We used IBM SPSS Statistics version 28 software for Mac.

### Ethical considerations

This study was approved by the Ethics Committee of the Graduate School of Human Sciences (Behavioral Sciences) at Osaka University (reference numbers: 27–017, 28–006). INTAGE HOLDINGS explained the purpose of the survey on the web. Participants were considered to have granted consent by responding to the survey.

## Results

### *Participants for analysis 1 (*Table 1*)*

Data were analyzed for 710 cancer patients who completed the Time1 survey. The mean time since diagnosis was 56.7 months (*SD* = 54.4; *range* = 3.5–398.5). Less than 85% of participants reported that they were either able to perform normal activities or had no complaints based on the KPS (Table 1). Based on the cutoff point of the HADS score, however, 43.5% of the participants (*n* = 309) might have suffered from adjustment disorder or major depression.

A total of 111 participants used psychosocial support services to cope with their distress or problems. The contents of these services are listed in Table2.

### Analysis 1: examining the support receiver and support non-receiver

We conducted *t*-tests to compare the PTGI scores between those who had used psychological support services (*n* = 111) and those who had never used such services (*n* = 599) at Time1 (Table 3). Support Receivers at the time of the Time1 survey reported significantly higher scores in the PTGI total score (*t* (708) = 2.032, *p* = .043, *d* = .210) and in Appreciation of Life, (*t* (708) = 3.274, *p* = .001, *d* = .338) and showed significant trends in New Possibilities (*t* (141.387) = 1.959, *p* = .052, *d* = .225).

**Table 3:** The difference of PTGI level between support receiver and support non-receiver

	Total		Support non-receiver		Support receiver		*t*	*p*	*Cohen’s d*
	(*N* = 710)		(*n* = 599)		(*n* = 111)				
	*M*	*SD*	*M*	*SD*	*M*	*SD*			
Relating to Others	1.85	1.06	1.83	1.04	2.00	1.18	1.438	.153	.162
New Possibilities	1.81	1.13	1.77	1.10	2.02	1.28	1.959	.052†	.225
Personal Strength	2.07	1.09	2.06	1.05	2.12	1.27	0.512	.609	.060
Spiritual Change	1.19	1.09	1.16	1.09	1.31	1.10	1.258	.209	.130
Appreciation of Life	2.50	1.13	2.44	1.11	2.82	1.17	3.274	.001**	.338
Total score of PTGI	1.91	0.95	1.88	0.92	2.08	1.04	2.032	.043*	.210

^**^p < .01, *p < .05, †*p* < .10

### *Participants for analysis 2 (*Table 1*)*

Data from 395 cancer patients who completed both the Time1 and Time2 surveys were analyzed. The mean time since diagnosis was 59.5 months (*SD* = 57.4; *range* = 3.5–398.5). More than 85% of those who participated at Time2 reported that they were either able to perform normal activities or had no complaints based on the KPS (Table 1). Based on the HADS score cutoff point, 40.3% of the participants (*n* = 159) might have suffered from adjustment disorder or major depression.

Of the 395 participants, 21 used psychosocial support services for the first time between the two surveys. The details of these services are presented in Table 2.

### *Analysis 2: PTG* × *utilization of psychological support services*

As a result of the correlation analysis among the 395 participants (Table 4), in Support Non-Receivers, Appreciation of Life (Time1 and Time2) was negatively correlated with age (*r* =  − .194, *p* < .001 and − .155, *p* = .003), and a similar trend was found in Support Receivers (*r* =  − .175, *p* = .449 and *r* = − .371, *p* = .098). There were significant positive correlations between sex and Personal Strength (Time2) in both groups (Support Non-Receivers* r* = .108, *p* = .036; Support Receivers *r* = .531, *p* = .013), meaning females reported a greater sense of Personal Strength than males. Additionally, there were positive correlations between sex and Appreciation of Life (Time1 and Time2) (*r* = .182, *p* < .001 and *r* = .158, *p* = .002) in Support Non-Receivers. Similar relationships were found in Support Receivers (*r* = .379, *p* = .091 and *r* = .605, *p* = .004), indicating that female patients reported a greater sense of Appreciation of Life than males.

**Table 4:** Correlation analysis of the PTGI at Time1 and Time2 by groups

Support receivers																Support Non-Receivers (*n* = 374)		Support Receivers (*n* = 21)	
Support Non-Receivers		1	2	3	4	5	6	7	8	9	10	11	12	13	14	*M*	*SD*	*M*	*SD*
1	Age(T1)	-	− .566**	.182	− .014	.009	.285	− .175	.078	.053	− .138	− .089	.210	− .371	− .080	59.2	11.3	52.2	15.2
2	Sex(T1; male = 1, female = 2)	− .559**	-	.171	.235	.335	− .115	.379	.261	.231	.310	.531*	.060	.605**	.415	1.5	0.5	1.5	0.5
3	Relating to Others T1	.038	.050	-	.716**	.741**	.580**	.464*	.931**	.787**	.504*	.441*	.502*	.304	.663**	1.8	1.0	2.1	1.1
4	New Possibilities T1	− .013	.056	.816**	-	.633**	.434*	.490*	.864**	.742**	.805**	.490*	.451*	.404	.757**	1.8	1.1	2.2	1.1
5	Personal Strength T1	− .012	.085	.691**	.755**	-	.592**	.405	.848**	.821**	.725**	.758**	.554**	.434*	.845**	2.1	1.0	2.4	1.0
6	Spiritual Change T1	− .002	.043	.546**	.508**	.458**	-	.045	.627**	.454*	.454*	.446*	.902**	.091	.534*	1.2	1.1	1.4	1.2
7	Appreciation of Life T1	− .194**	.182**	.586**	.636**	.579**	.381**	-	.599**	.523*	.505*	.377	.088	.771**	.578**	2.5	1.0	3.3	1.1
8	Total score of PTG T1	− .025	.089	.926**	.927**	.848**	.633**	.743**	-	.864**	.745**	.609**	.588**	.481*	.843**	1.9	0.9	2.3	0.9
9	Relating to Others T2	.068	.024	.689**	.629**	.511**	.399**	.509**	.685**	-	.754**	.573**	.489*	.477*	.876**	1.8	1.1	2.1	1.2
10	New Possibilities T2	.012	.057	.586**	.687**	.545**	.388**	.517**	.670**	.843**	-	.753**	.506*	.625**	.926**	1.8	1.2	2.4	1.3
11	Personal Strength T2	.015	.108*	.548**	.586**	.623**	.331**	.446**	.626**	.750**	.779**	-	.508*	.605**	.840**	2.0	1.1	2.4	1.3
12	Spiritual Change T2	.030	.020	.468**	.495**	.384**	.674**	.389**	.547**	.617**	.669**	.520**	-	.298	.624**	1.2	1.2	1.4	1.3
13	Appreciation of Life T2	− .155**	.158**	.479**	.465**	.432**	.296**	.668**	.556**	.627**	.644**	.529**	.470**	-	.713**	2.5	1.1	3.1	1.2
14	Total score of PTG T2	.010	.077	.669**	.684**	.590**	.453**	.583**	.729**	.940**	.942**	.857**	.718**	.744**	-	1.9	1.0	2.3	1.0

^**^p < .01, *p < .05

*T1*, Time1; *T2*, Time2

To examine the relationship between PTG and HSB, we conducted mixed ANOVAs for PTG (Time1, Time2) and HSB (the utilization of psychological support services). The results showed no significant interaction or main effect of time (Table 5). There were significant main effects of HSB on the total PTG score (*F* (1, 393) = 4.023, *p* = .046, partial *η*^*2*^ = .010) and two of the PTG domains; Appreciation of Life (*F* (1, 393) = 10.811, *p* = .001, partial *η*^*2*^ = .027) and New Possibilities (*F* (1, 393) = 4.284, *p* = .039, partial *η*^*2*^ = .011).

**Table 5:** Results of a mixed ANOVA for PTG and HSB

	Time1						Time2						Main effect							Interaction effect	
	Support Non-Receivers (*n* = 374)			Support Receivers (*n* = 21)			Support Non-Receivers (*n* = 374)			Support Receivers (*n* = 21)			Time			Use					
	*M*	*SD*		*M*	*SD*		*M*	*SD*		*M*	*SD*		*F*	*p*		*F*	*p*			*F*	*p*
Relating to Others	1.84	1.02		2.06	1.08		1.84	1.08		2.15	1.18		0.218	.641		1.524	.218			0.242	.623
New Possibilities	1.81	1.07		2.21	1.12		1.82	1.15		2.37	1.30		0.798	.372		4.284	.039	*		0.565	.453
Personal Strength	2.08	1.04		2.42	1.00		2.05	1.11		2.40	1.30		0.036	.850		2.546	.111			0.006	.941
Spiritual Change	1.16	1.10		1.36	1.22		1.23	1.16		1.43	1.28		0.471	.493		0.695	.405			0.001	.982
Appreciation of Life	2.48	1.05		3.29	1.08		2.47	1.11		3.13	1.23		0.716	.398		10.811	.001	**		0.585	.445
Total score of PTG	1.90	0.90		2.27	0.89		1.91	0.97		2.32	1.04		0.116	.733		4.023	.046	*		0.095	.758

^**^p < .01, *p < .05

### Analysis 3: relationship between PTG and the utilization of psychological support services

Hierarchical logistic regression analyses were conducted to examine whether experiencing PTG (Time1) may lead to HSB (Time2). The results showed that age (*OR* = .946, *CI* = .905–.989, *p* = .014) and Appreciation of Life at Time1 (*OR* = 1.954, *CI* = 1.249–3.059, *p* = .003) were significantly related to HSB usage (Table 6).

**Table 6:** PTG domains associated with the use of psychosocial support services

	*OR*		*CI*
*Step1*			
Age	.946	*	.905–.989
Sex (male = 0, female = 1)	.447		.156–1.286
*Step2*			
Appreciation of Life	1.954	**	1.249–3.059

^**^p < .01, *p < .05

*Nagelkerke R2* = .129

Hosmer–Lemeshow test *p* = .673

Other four factors of the PTGI were removed from this model

## Discussion

This study aimed to examine the complementary relationship between PTG and HSB using a longitudinal research design among patients with cancer.

### Experiencing HSB may lead to PTG

Analysis 1 revealed that those who experienced HSB at Time1 had higher total PTGI scores. Analysis 2 showed only a main effect of HSB, which meant that using psychosocial support services did not increase the level of PTG score in the setting of this study. In summary, this study partially supports a previous study [34] and the hypothesis that PTG is higher when engaging in HSB. It is possible that PTG is not high, either because it requires time to be experienced and recognized or because patients perceive that they are in a state in which it is not a problem to not use it now in Support Receivers. Those who used such services were presumably still in a difficult situation, so their PTG was not high enough to be significantly different from that of Support Non-Receivers. Furthermore, one report suggested that there were two groups of people who did not intend to use psychosocial support services among Support Non-Receivers: one group may have adjustment disorder or major depression, and the other does not, people in the latter were high extroverted [39] and thus more likely to experience PTG [11]. Therefore, no significant interaction effect may have been found. In this study, psychosocial support services varied in terms of content, length of time since the beginning of use of each service, and the way in which the services were related to the patients. A study examining the impact of psychosocial intervention on PTG also observed that the results may become less robust when there is diversity of services [31]. In addition, it is important to note that Analysis 1 did not examine within-individuals, as it focused only on the cross-sectional design at Time1.

In this study, people engaged in HSB were likely to report higher levels of PTG, especially for the Appreciation of Life and New Possibilities of the PTGI. It may be that the person’s priorities in life have changed due to using the support, or it may be that Appreciation of Life in general has emerged as the person tries to cope with things while using the support. Additionally, the use of support might have led some people to interact with people they would not have met if they had not been diagnosed with cancer (e.g., psychotherapists and patients with the same disease), which might have led to new interests. Meanwhile, for some people, cancer may have limited their possibilities and prevented them from feeling a strong sense of New Possibilities, which may offset the strong group differences.

### Experiencing PTG may lead to HSB

Based on Analysis 2, participants engaged in HSB may have reported higher PTG, especially regarding Appreciation of Life, to begin with. In addition, Analysis 3 showed that this domain may lead to HSB. These results support our hypothesis and provide a new finding that has not been examined in previous studies. That is, as one previous study [8] suggested, Appreciation of Life would have the characteristics that having survived the traumatic event, people felt better to cope with future challenging situations, and thus, as this component increase, patients with cancer may tend to use psychosocial support services as a coping such challenging situation. Alternatively, having cancer may have made participants aware of the importance of their own lives or it might have changed their priorities in life, which turned them to using psychosocial support services to help them adapt to their “new normal.” The experience of cancer may have increased general feelings of Appreciation of Life, which may have increased the likelihood of HSB due to gratitude and a greater willingness to be vulnerable with others. This would make it easier for patients to accept HSB recommendations from the medical staff, which has been reported to be an important promoter [18].

Given the contents of the PTGI items assessing Relating to Others domain (e.g., “I am more willing to express my emotions,” “I more clearly see that I can count on people in times of trouble.”), we hypothesized that patients with a high level of this domain of PTG would use more services. Although Support Receivers in Time2 showed a higher level of this PTG domain (Table 5), no statistical relationships were found in Analysis 1 or the hierarchical logistic regression model in Analysis 3. This may be because patients with cancer who participate in a group problem-solving therapy generally report more interpersonal problems [40], which might have a confounding impact on service usage. Moreover, there may be a matter of factor structures. Although the present study adopted the original five factors, one of the items in the Relating to Others factor (“I learned a great deal about how wonderful people are.”) was once reported to be included in the Spiritual Change and Appreciation of Life factor with a Japanese sample [22].

### Complementary relationships between PTG and HSB

Overall, our hypotheses were supported by showing the two-way relationship. Those who received psychosocial support services reported higher PTG. It is also possible that people engage in the HSB, because they experienced PTG. This suggests that people who were likely to seek help for psychosocial support services and those who had experienced PTG had common characteristics. Previous studies have examined trajectories of PTG after cancer diagnosis or surgery and found several patterns [12–14]. One study reported that the group with elevated PTG during the course of the study had greater use of active-adaptive coping strategies, including active coping and emotional support, at base line [13]. Without such characteristics, people might not fit the existing PTG or HSB model. The results of this study confirm the arguments of previous studies [11]. It is not expected that all cancer survivors will experience PTG, nor is it necessary to use psychosocial support services. Approaches that promote conformity to existing systems are important and have been studied [19, 33]. However, as this study suggests, some people may not adapt to them. This does not mean that such people do not experience PTG or need help. As there would be PTG for such people and support that would be acceptable to them, care or support should be provided while taking this direction into consideration.

As mentioned, the PTGI-X [23] has been developed to expand the component of the existing PTGI, which would be able to capture cancer patients’ self-reported growth in a wider array of cultural contexts, such as having a greater sense of harmony and connectedness. In addition, there are reports on “growth” that have not been fully captured in the previous PTGI [8, 41, 42]. A review of patients with cancer mentioned that previous studies might not completely capture the entirety of positive responses among them [5], and one study reported the contents of PTG specific to cancer patients [43]. People who do not fit the existing model are presumed to be highly introverted [11]. Studies investigating barriers to the use of psychosocial support services found that concerns about interpersonal interactional burden or unwillingness to talk about their own experiences prevented their use [16, 44]. It may also be important to provide support services that solve the problems of cancer patients with little or no interpersonal interaction.

## Limitations

This study has several limitations. First, there were biases regarding the participants in terms of using the Internet; therefore, there might have been a selective bias. Second, there were variations in terms of the contents of psychosocial support services and the duration of use. In this study, we did not limit the types of available psychosocial support services. The duration of use for some types of services may not have been long enough to increase PTG levels. The sample sizes were unequal, with that of Support Receivers being small, as the number of users in Japan tends to be small and because the patients might be receiving informal support. Furthermore, the causation remains unknown, as we cannot determine whether one causes the other. Despite these limitations, we have generally demonstrated a complementary relationship between the two concepts, PTG and HSB.

## Conclusion

This study examined the hypothesis that PTG and HSB are mutually related, which is generally supported. These results suggest that the two may have common features. Medical staff should encourage patients to seek psychosocial support; as for some people, it could promote PTG or vice versa. At the same time, however, it is important to acknowledge that not all patients should experience PTG or be willing to receive professional psychosocial support.

## Data Availability

Not applicable.
